# 5-year migration and inducible displacement of the uncemented LCS and ATTUNE rotating platform knee systems: a secondary report of a randomized controlled RSA trial

**DOI:** 10.2340/17453674.2024.42744

**Published:** 2025-01-10

**Authors:** Raymond PUIJK, Lennard A KOSTER, Bart G C W PIJLS, Jiwanjot SINGH, Marjolein SCHAGER, Bart L KAPTEIN, Peter A NOLTE

**Affiliations:** 1 Department of Orthopaedics, Spaarne Gasthuis, Hoofddorp; 2 Department of Orthopaedics, Leiden University Medical Center, Leiden; 3 Department of Oral Cell Biology, Academic Centre for Dentistry (ACTA), University of Amsterdam and Vrije Universiteit Amsterdam, Amsterdam, the Netherlands

## Abstract

**Background and purpose:**

Early migration of the uncemented cruciate-sacrificing rotating platform ATTUNE and Low Contact Stress (LCS) tibial components was classified as at-risk for aseptic loosening rates exceeding 6.5% at 15 years based on recent fixation-specific migration thresholds. In this secondary report of a randomized controlled trial (RCT) we aimed to evaluate whether the 5-year migration, inducible displacement, and the clinical outcome of the ATTUNE components were comparable to those of the LCS.

**Methods:**

Patients from the initial 2-year radiostereometric analysis (RSA) RCT were recruited for a 5-year follow-up. At 5 years, participants underwent 2 supine and 1 loaded RSA examination, clinical assessments, and questionnaires. Migration was analyzed using maximum total point motion (MTPM), translations, and rotations, focusing on 5-year migration, continuous migration (> 0.10 mm/year), and inducible displacement. Revisions, along with clinical and functional outcomes, were also evaluated.

**Results:**

At 5 years, 24 ATTUNE and 24 LCS implants were analyzed. The mean MTPM was similar for tibial components (ATTUNE 1.13mm [confidence interval (CI) 0.94–1.33]; LCS 1.24 mm [CI 1.05–1.46]) but significantly lower for the ATTUNE femoral component (1.14 mm [CI 0.92–1.39]) than LCS 1.87 mm [CI 1.57–2.21]). 2-to-5-year migration rates were comparable, but 11 ATTUNE and 7 LCS exceeded 0.10 mm MTPM/year, indicating a higher risk of loosening. Inducible displacement was similar, although 1 patient with a tibial ATTUNE showed excessive displacement (3.34 mm MTPM) with focal osteolysis but no symptoms. 1 revision 10 days post-surgery was performed for an ATTUNE insert spinout, resolved with an isolated insert exchange. Clinical and functional outcomes were comparable.

**Conclusion:**

At the 5-year follow-up, ATTUNE tibial components showed similar migration, while the femoral component migrated significantly less than the LCS, which mainly occurred during the first 2 years. 2-to-5-year migration rates, inducible displacement, and clinical and functional outcomes were comparable. These findings suggest a comparable long-term risk of aseptic loosening between the uncemented ATTUNE and LCS knee systems.

The introduction of new total knee replacements (TKRs) or fixation methods carries the potential risk of higher revision rates, with several examples of newer implants showing inferior results compared with their predecessors [1-4]. A prominent case is the original cemented ATTUNE knee system from 2012 (DePuy Synthes, Warsaw, IN, USA), which exhibited a higher failure rate than earlier designs due to debonding at the tibial implant–cement interface [5,6]. To address this, the ATTUNE S+ design was introduced in 2017, incorporating an improved “macrolock” cement-locking mechanism and “microblast” surface, to enhance stability [5,6]. For this reason, it remains crucial to closely study performance at an early stage, while wide usage of the implants is still limited.

Radiostereometric analysis (RSA) is a method that can accurately measure the 3-dimensional micromotions of an implant relative to the bone, requiring only a small number of patients to study [7]. When measured over time, implant migration patterns can provide valuable insight on the implants’ long-term quality of fixation and potential risk of loosening up to 15 years [8,9]. Additionally, inducible displacement is an RSA method that assesses the current state of the implant–bone fixation, by comparing its position under loading conditions, such as standing, with its position in an unloaded condition, e.g., non-weightbearing supine position [8,10-15]. In our previously performed RSA randomized controlled trial (RCT), we analyzed the 2-year migration of the newly introduced uncemented ATTUNE TKR and its predecessor, the uncemented Low Contact Stress (LCS) implant (DePuy Synthes, Warsaw, IN, USA) [16]. The study revealed similar improvements in patient-reported outcome measures (PROMs), a significantly lower femoral component migration of the ATTUNE compared with the LCS, and comparable migration patterns for both tibial components. However, according to the newly defined fixation-specific migration thresholds by Puijk et al. (2024), the 6- and 12-month migrations of both tibial components would be identified as at-risk for a revision rate higher than 6.5% due to aseptic loosening at 15 years [17]. While the uncemented LCS implant has been used for several decades, demonstrating excellent long-term revision rates for aseptic loosening [18-20], the uncemented ATTUNE system, first introduced in 2016, does not yet have similarly reassuring long-term data. This is a secondary report of an RCT aiming to investigate whether the uncemented ATTUNE TKR exhibits a comparable migration pattern to the LCS system in patients with end-stage primary osteoarthritis over a 5-year follow-up period. Secondarily, it compares the inducible displacement, clinical, and functional outcomes between implants.

## Methods

### Trial design

This follow-up study assesses the 5-year follow-up data, extending the analysis of a prior 2-year follow-up, single-center, single-blinded, noninferiority RCT in the Netherlands [16]. The conduct and reporting of this study adhere to the RSA (2024) [7] and CONSORT (2010) guidelines [21]. The RSA item checklist is provided in Table S1 (see Supplementary data).

### Participants

All patients with primary osteoarthritis who participated and underwent arthroplasty by 3 surgeons in the original study between July 2017 and April 2019 were deemed eligible. Patients were excluded if they had undergone major revision (exchange of the tibial or femoral component), or if they were not able or unwilling to participate. No exclusions were conducted when patients passed the recommended ±4 weeks examination window [7], Data was collected in the Spaarne Gasthuis Hospital (Hoofddorp, the Netherlands) and migration data further analyzed in the Leiden University Medical Center (Leiden, the Netherlands).

### Intervention, randomization, and blinding

Patients were randomly assigned to receive either uncemented cruciate-sacrificing rotating platform LCS or ATTUNE TKR in a 1:1 allocation ratio. Detailed information on the design differences, randomization, allocation, surgical techniques, and postoperative care is stated in the initial study (Koster et al. 2023) [16]. Patients were unblinded to the type of implant they received after 2 years. At the 5-year follow-up, the researchers (RP, JS) who performed the clinical assessments were blinded to the type of implant each patient had received.

### Radiostereometric technique

At the 5-year follow-up, available patients underwent 2 sequential non-loaded supine examinations and 1 loaded single-leg stance examination. The first supine examination was used for the migration pattern, while the second supine examination was used to calculate the measurement error (i.e., precision). Inducible displacement was calculated by comparing the displacement between the first supine and the loaded examination. For the single-leg stance, patients were instructed to fully bear their bodyweight on the operated leg, placing the contralateral foot on a diagonally positioned chair in front of them with their knee bent at a 90° angle.

Radiographs were taken by use of 2 DX radiology detectors (35 × 43 cm, Siemens Healthcare, Germany, and Carestream Health, USA) and an acrylic biplanar calibration cage (Baat Medical, Netherlands). Baseline reference examinations were undertaken on the first postoperative day after weightbearing. Migration was expressed using maximum total point motion (MTPM) and translations measured in millimeters (mm), and rotations in degrees (°). Migration of implants was measured using the right knee as the reference side. Positive translations were defined as proximal translation in the y-axis, medial translation in the x-axis, and anterior translation in the z-axis. Positive rotation directions were defined as internal rotation about the y-axis, anterior tilt about the x-axis, and varus rotation about the z-axis. Component migration was calculated using the consistent-marker method, by use of model-based RSA software (v.4.2, RSAcore, Leiden, Netherlands), utilizing computer-aided design models. The migration of previous follow-ups was recalculated by the same researcher (LAK) to ensure the same markers were used over the 5-year period. Condition numbers were limited to 120, and the mean error of rigid body fitting was restricted to 0.35 [7].

### Outcome measures

The primary outcome measure of this follow-up study was the 5-year migration pattern (MTPM) of both components and implants. The secondary outcome was the inducible displacement and clinical and functional outcome. For the clinical outcome, all complications up to 5 years were recorded. The functional outcome was measured by range of motion and stability of the joint. PROMs were collected by questionnaires, including the Oxford Knee Score (OKS), KOOS-PS, the Anterior Knee Pain Scale (KUJALA), EQ-5D-3L, the numeric rating scale for pain at rest (NRS-rest) and activity (NRS-activity) [16]. The KUJALA score was introduced for additional assessment of PROM outcomes after the 2-year report [16].

### Sample size

A sample size calculation for the initial 2-year RCT determined that 26 total knee replacements (TKRs) per group were necessary to confirm the non-inferiority of the ATTUNE system, with 80% power (α = 0.01, SD = 0.6 mm) [16].

### Statistics

All migration and displacement values were presented as means and 95% confidence intervals (CI). MTPM values were log-transformed (log10) for analyses and back-transformed for presentation in Tables and Figures. All repeated migration measures were calculated by use of a linear mixed-effects model (LMM). The number of implants that had a higher 2- to 5-year migration than the 0.1 mm/year threshold were evaluated, as this has been suggested to be associated with a higher risk of loosening, according to Ryd et al. [22]. Measurement error statistics from double examinations at 5 years were provided for each migration direction [7]. The mean inducible displacement at 5 years was compared with this measurement error to assess actual inducible displacement. The inducible displacement of cases with a greater than 0.30 mm (> 0.1 mm/year) 2- to 5-year migration was evaluated. The distribution of the CIs of migration values was compared to evaluate significance.

The differences in clinical and functional outcomes were evaluated for clinical relevance, and presented in boxplot diagrams alongside the previous interval scores. Analyses were performed using R software (v.4.2.1, R Foundation for Statistical Computing, Vienna, Austria).

### Sensitivity analysis

Sensitivity analyses were conducted to evaluate whether patients outside ±4 weeks examination window or using the second instead of the first supine examination at 5 years altered results. The potential influence of attrition bias was assessed by comparing the 2-year migration patterns for patients with and without 5-year follow-up.

### Ethics, registration, data sharing, funding, and disclosure

The Committee for Medical Ethics of Leiden University Medical Center (LUMC) approved the study (Protocol ID P22.065, ABR NL82000.058.22) and the study was registered in Clinical Trials (ClinicalTrials.gov ID NCT05623215). All patients provided informed consent. Data is available by contacting the corresponding author. Both the original and current studies were investigator-initiated. In the original trial, DePuy Synthes provided funding for the radiostereometric analysis. In the current study, no external funding was received, and DePuy Synthes played no role in the study’s design, analysis, interpretation, or manuscript preparation. No author had any conflicting interest. Complete disclose of interest forms according to ICMJE are available on the article page, doi: 10.2340/17453674.2024.42744

## Results

**Figure 1:**
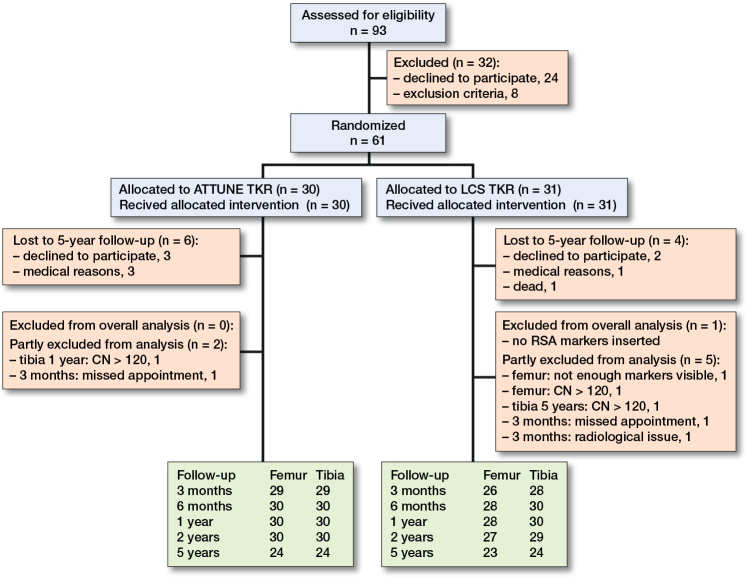
Flow diagram of patients through each stage of the study. CN: condition number; LCS: Low Contact Stress; RSA: radiostereometric analysis; TKA: total knee arthroplasty.

24 LCS (14 females) and 24 ATTUNE (11 females) uncemented TKR systems were included (Figure 1). For the patients available at the 5-year follow-up, the ATTUNE group had a mean age of 72.6 years (SD 8.6) and BMI of 30.1 (SD 5.3), while the LCS group had a mean age of 73.2 years (SD 6.3) and BMI of 27.6 (SD 2.6) at the 5-year follow-up. The mean follow-up duration was 61.3 months (SD 3.1). 8 patients were examined outside the recommended ±4 weeks window, with a follow-up range of 59–73 months. The systematic measurement error (precision) of the double examinations at 5-years were similar for the 2 systems (Table 1).

**Table 1:** Values of measurement error statistics (i.e., precision) of both components of the uncemented ATTUNE and LCS total knee system based on double examinations at 5-years postoperatively. Values are mean (standard deviation)

Component	Tx, mm	Ty, mm	Tz, mm	Rx, °	Ry, °	Rz, °	MTPM, mm
Femoral component							
ATTUNE	–0.04 (0.12)	0.01 (0.05)	–0.07 (0.43)	0.07 (0.41)	–0.02 (0.14)	–0.07 (0.17)	0.30 (0.63)
LCS	0.00 (0.05)	–0.01 (0.04)	–0.01 (0.08)	–0.02 (0.12)	0.00 (0.16)	–0.06 (0.10)	0.22 (0.12)
Tibial component							
ATTUNE	0.02 (0.06)	0.00 (0.03)	0.01 (0.05)	0.01 (0.07)	–0.04 (0.20)	–0.01 (0.11)	0.18 (0.11)
LCS	–0.01 (0.04)	0.01 (0.04)	0.03 (0.08)	0.00 (0.17)	0.01 (0.34)	0.00 (0.10)	0.27 (0.17)

Rx, Ry, and Rz represent rotations around, and Tx, Ty, and Tz represent translations along the x-, y-, and z-axes. For a right-sided knee, positive translations are medial (x), proximal (y), and anterior (z) movements; positive rotations (°) are anterior tilt about the x-axis, internal rotation about the y-axis, and varus rotation about the z-axis.

LCS: Low Contact Stress; MPTM: maximal total point motion.

### Migration pattern

#### Tibial components

**Figure 2:**
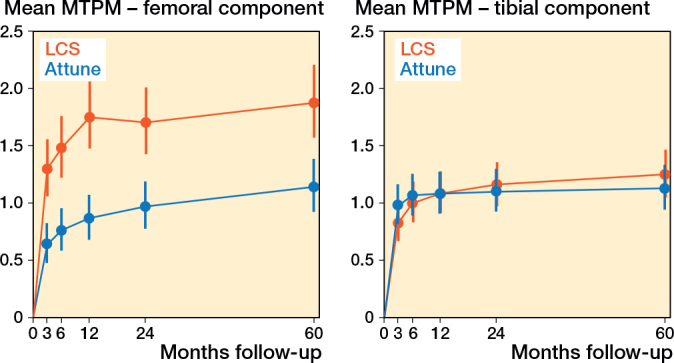
Postoperative time after surgery versus longitudinal mean migration patterns of the Attune and LCS femoral components, measured by maximum total point motion (MTPM) in millimeters. Migration was derived from the linear-mixed model analyses. Error bars indicate 95% confidence intervals.

The mean MTPM was 1.13 mm (CI 0.94–1.33) and 1.24 mm (CI 1.05–1.46) for the ATTUNE and LCS after 5 years, respectively (Figure 2). The LCS translated significantly in lateral, distal, and posterior directions, and rotated counterclockwise for any direction at 5 years compared with baseline (Table S2, Figure S1, see Supplementary data). The ATTUNE showed a significant distal translation at 5 years compared with baseline. The CIs of all directions were deemed comparable at the 5-year examination mark (Table S2, see Supplementary data). Between 2 and 5 years, no significant migration was detected in any direction, with similar outcomes observed for both components (Table 2). 4 ATTUNE and 4 LCS tibial components migrated more than 0.30 mm (> 0.1 mm MTPM/year) between 2 and 5 years postoperatively (Figure 2). 1 of the 4 ATTUNE components showed a clear focal osteolysis on the lateral and posterior side of the component, without any complaints of pain (NRS of 0 in rest, and 1 during movement) (Figure S4, see Supplementary data). Other implants had no obvious reasons for their increased continuous migration.

**Table 2:** Differences in 2- to 5-year migration between components of the ATTUNE and LCS prostheses. Mean values with 95% confidence intervals (CI), as derived from the linear mixed-effects model

Factor	ATTUNE mean (CI)	LCS mean (CI)
Femoral component		
Tx, mm	0.05 (–0.10 to 0.21)	–0.02 (–0.18 to 0.14)
Ty, mm	0.02 (–0.11 to 0.16)	0.05 (–0.09 to 0.19)
Tz, mm	–0.02 (–0.32 to 0.28)	–0.10 (–0.41 to 0.21)
Rx, °	0.05 (–0.33 to 0.42)	0.16 (–0.23 to 0.55)
Ry, °	0.00 (–0.29 to 0.29)	–0.15 (–0.45 to 0.15)
Rz, °	0.10 (–0.22 to 0.42)	–0.06 (–0.39 to 0.28)
MTPM, mm	0.20 (–0.26 to 0.67)	0.22 (–0.26 to 0.70)
Tibial component		
Tx, mm	–0.01 (–0.14 to 0.12)	–0.01 (–0.14 to 0.12)
Ty, mm	0.03 (–0.15 to 0.21)	0.05 (–0.13 to 0.24)
Tz, mm	–0.01 (–0.19 to 0.17)	0.01 (–0.17 to 0.19)
Rx, °	0.07 (–0.33 to 0.48)	–0.04 (–0.46 to 0.36)
Ry, °	–0.07 (–0.33 to 0.19)	–0.23 (–0.49 to 0.03)
Rz, °	–0.03 (–0.26 to 0.20)	–0.08 (–0.31 to 0.14)
MTPM, mm	0.02 (–0.27 to 0.30)	0.13 (–0.16 to 0.42)

For abbreviations, see Table 1.

#### Femoral components

**Figure 3:**
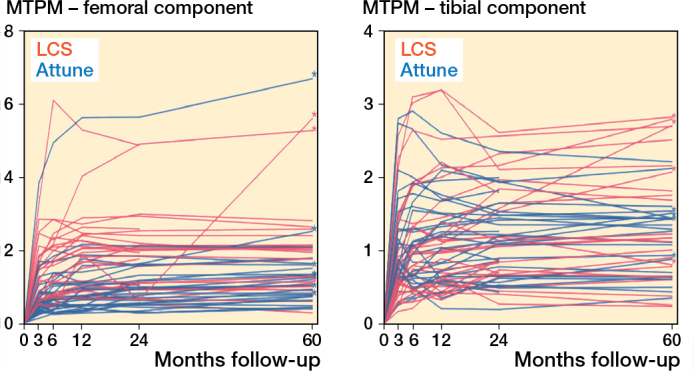
Longitudinal individual 5-year migration patterns of the Attune and LCS femoral and tibial components, measured by maximum total point motion (MTPM) in millimeters. * A 2- to 5-year migration (MTPM) of more than 0.1 mm/year (0.3 mm).

At 5 years postoperatively, the ATTUNE had a lower mean MTPM of 1.14 mm (CI 0.92–1.39) than the 1.87 mm (CI 1.57–2.21) of the LCS femoral component (Figure 2). Additionally, the ATTUNE translated less proximally (0.27 mm [CI 0.17–0.37] vs 0.77 mm [0.67–0.88]), and had less external rotation (–0.24° [CI –0.45 to –0.03] vs –0.70° [CI –0.92 to –0.49]), compared with the LCS component at 5 years (Table S2, Figure S2, see Supplementary data). Between 2 and 5 years postoperatively, no significant migration was observed in any direction, with comparable results for both components (Table 2). A total of 7 ATTUNE and 3 LCS implants had a larger 2- to 5-year migration (MTPM) than 0.30 mm (Figure 3), which would indicate a higher risk of loosening according to the 0.1 mm/year thresholds. In 1 femoral LCS component, a migration (MTPM) of 4.99 mm was measured between 2 and 5 years, which was attributed to a lateral femoral condyle fracture, 4 months prior to the 5-year examination. This patient presented no complaints of pain (NRS of 0 in rest, and 1 during movement). Other implants showed no clear reason for their increased continuous migration.

Sensitivity analyses showed no differences in mean migration results when excluding cases with their 5-year examination outside the ±4 weeks window. The 2-year mean migration patterns suggested an attrition bias, with both LCS components without a 5-year follow-up showing slightly higher MTPM compared with other groups with and without 5-year follow-up (Figure S3, see Supplementary data).

### Inducible displacement

No differences in inducible displacement (MTPM) and corresponding CIs were observed between the femoral and tibial components of both implants (Table 3). The mean inducible displacements (MTPM) of all components were larger than the measurement errors from the double examinations at 5-year follow-up, although all had overlapping CIs (Table 3). Detailed inducible displacement data, including the translations and rotations, are provided in Table S3 (see Supplementary data).

**Table 3:** Inducible displacement and the measurement error at 5 years postoperatively, between the ATTUNE and LCS components. Values are mean MTPM (in mm) and (CI)

Component	n	Inducible displacement	Measurement error (supine doubles)
Femoral component			
ATTUNE	24	0.28 (0.21–0.35)	0.23 (0.17–0.30)
LCS	22	0.30 (0.23–0.36)	0.27 (0.18–0.36)
Tibial component			
ATTUNE	24	0.37 (0.22–0.53)	0.24 (0.16–0.32)
LCS	24	0.39 (0.29–0.51)	0.30 (0.25–0.35)

MTPM: maximal total point motion; mm: millimeter; CI: 95% confidence interval.

For the ATTUNE implant, the femoral component showed a significant displacement along the y-axis of 0.03 mm (CI 0.01–0.04), and –0.05 mm (CI –0.07 to –0.03) for the tibial component. For the LCS femoral component, a significant posterior translation along the z-axis of –0.03 (CI –0.06 to –0.01) was observed compared with the measurement error (Table S3, see Supplementary data). When only the inducible displacement of components with > 0.30 mm (MTPM) 2–5-year migration rate was considered, the mean displacement was 0.21 mm (SD 0.07) for the 7 femoral ATTUNE, 0.19 mm (SD 0.02) for the 3 femoral LCS, 1.01 mm (SD 1.50) for the 4 tibial ATTUNE, and 0.29 mm (SD 0.04) for the tibial 4 LCS component. Of these tibial ATTUNE components, 1 had an inducible internal rotation of 4.73° and MTPM of 3.34 mm. Additionally, the patient who suffered from a lateral femoral condyle fracture 4 months before the examination had an inducible displacement of 0.11 mm MTPM.

A sensitivity analysis showed that when the second supine examination at 5 years was used as a reference, no relevant differences were observed compared with the original analyses.

### Clinical outcomes

There were no differences between the 2 components in clinical and functional outcomes at 5 years (Table S4 and S5, see Supplementary data). The KUJALA score, added additionally after the 2-year report (16), was 76.9 (SD 15.3) for the ATTUNE and 79.9 (SD 17.6) for the LCS after 5 years, indicating similarity. From 2 to 5 years, the mean scores for OKS, KOOS-PS, EQ-5D-3L, and NRS (rest and activity) were comparable and showed no further improvement. One ATTUNE insert spinout occurred 10 days post-arthroplasty in a patient with valgus deformity and was revised to a thicker insert, with no further issues reported thereafter. Another patient with an LCS had a femoral lateral condyle fracture 4 months before the 5-year RSA exam, treated with a plaster sleeve and hinged brace for 12 weeks. The 5-year migration data of both patients was included in the analyses.

## Discussion

In this follow-up RCT we aimed to evaluate whether the 5-year migration, inducible displacement, and clinical outcome of the ATTUNE components were comparable with those of the LCS. We observed comparable mean 5-year migration of the tibial components in all directions. However, the ATTUNE femoral components showed less migration at 5 years compared with the LCS components. This difference was primarily attributed to the greater proximal translation and external rotation observed in the LCS femoral components. The higher averages of both directions were largely influenced by 2 LCS components: 1 showed substantial proximal and anterior translation, posterior tilt, and external and valgus rotation after 2 years but stabilized by 5 years; the other experienced a significant proximal and posterior translation, anterior tilt, and external rotation due to trauma 4 months before the 5-year examination. Additionally, the mean 2- to 5-year migration rate showed no clinically relevant differences between the components, despite the 11 ATTUNE (4 tibia) and 7 LCS (4 tibia) components that had a higher rate of 0.30 mm (> 0.1 mm MTPM/year).

To date, there are no other studies comparing the migration patterns of the ATTUNE and LCS knee systems beyond this and the previous study, making it difficult to compare our results with other research. However, the migration patterns of both tibial components fell within the 25th to 75th percentile range for all uncemented implants, and for similar porous-coating or mobile-bearing characteristics, as determined by a meta-analysis of RSA studies up to 2023 [9]. Recent findings indicate that relying on the mean continuous migration of study groups is not valuable for estimating the collective risk of revision of aseptic loosening, and thus also not for use in a phased introduction of implants [17]. However, by employing an RCT design, this study is optimally positioned to enable reliable conclusions regarding comparability in fixation, encompassing both early migration and 5-year inducible displacement. Additionally, considering excellent long-term revision rates established by the uncemented LCS system, there is a strong likelihood that the uncemented ATTUNE system will exhibit comparable performance in this regard. For instance, the Dutch Arthroplasty Register (LROI) recorded a major revision rate (e.g., not exclusively aseptic loosening) of 3.3% (CI 2.8–3.7) for 9,032 uncemented LCS implants after 14 years of follow-up [20]. Similarly, the NRJ registry reported a revision rate of 4.0% (CI 3.6–4.4) for 16,494 LCS knees [23]. However, the Australian registry reported a higher all-cause revision rate of 7.3% (CI 6.2–8.5) for 2,379 LCS knees after 15 years, without specific reasoning known [24]. As for the uncemented ATTUNE system, the LROI reported a revision rate of 1.4% (CI 0.0–3.1) for 467 implants, and the Australian registry reported a rate of 1.2% (CI 0.5–2.8) for 1,632 implants after 3 years of follow-up [20,24]. Although no long-term follow-up revision rates for the ATTUNE system are yet available, the mean rates at 3 years are so far lower than those known for the LCS at the same interval: 2.2% (CI 1.8–2.5) (LROI), 1.8% (CI 1.6–2.0) (NJR), and 3.4% (CI 2.7–4.2) (Australia) [20,23,24].

In the current study, the mean inducible displacement was similar to that observed in other uncemented implants from studies with comparable long-term follow-up [8,13]. The 10-year follow-up study by Laende et al. (2019) reported a significantly lower mean displacement of 0.18 mm (SD 0.18) in 21 uncemented implants compared with 0.38 mm (SD 0.37) in cemented implants [8]. Similarly, Wilson et al. (2010) found a displacement of 0.19 mm (SD 0.06) in trabecular metal uncemented implants compared with 0.34 mm (SD 0.13) in cemented implants [13]. Both studies suggesting comparable or better fixation of uncemented implants at the time of inducible displacement measurement [8,13]. While the utility of inducible displacement shows great promise for detecting loosened implants, its role in risk assessment during phased implant introduction remains uncertain [8,11-14]. In the current study, 1 ATTUNE tibial component with substantial inducible displacement showed a significant post hoc risk for aseptic loosening [7], given the increased prior probability due to the presence of focal osteolysis (Figure S4, see Supplementary data). Nevertheless, the patient has not presented any symptoms yet, and therefore no revision has been performed. Notably, this tibial component was 1 of 18 components with continuous migration of 0.10 mm/year, which would suggest a higher risk of loosening for more implants. One possible explanation, which we propose, is that uncemented implants may possess the ability to re-establish bone ingrowth and fixation even after an initial loosening event. This hypothesis is supported by our study’s findings, where the LCS femoral component, which exhibited a migration rate of 4.99 mm (MTPM) between 2 and 5 years following a distal femoral fracture sustained 4 months earlier, demonstrated only an inducible displacement of 0.11 mm at the 5-year follow-up, with no reports of pain in the PROM assessment. However, further research would be needed to confirm this hypothesis. In the study by Lam Tin Cheung et al. (2018), which included 15 cemented implants followed up for 10 years, no correlation was found between inducible displacement and the 2- to 10-year migration rate [14]. A longer follow-up of this trial would be required to determine whether the components with higher migration rates and inducible displacement could re-achieve fixation.

### Limitations

First, a notable proortion of patients were lost to follow-up, primarily due to medical reasons (n = 5) or unwillingness to participate (n = 5). A sensitivity analysis to assess the impact of this attrition bias showed slightly higher migration at 2 years for the group without a 5-year follow-up. Although this indicates some attrition bias, the minimal difference in migration and non-overlapping CIs suggest that this bias likely did not influence our conclusions. Nevertheless, the 5-year LCS migration data would likely be slightly higher without any attrition bias.

Second, the researcher (LAK) who performed the RSA measurements was aware of the TKR design during analyses, thus these measurements were not blinded. However, RSA measurements are inherently objective rather than subjective, so it is expected that this would not lead to different results. Third, this study did not include a point motion analysis of fictive points, which could have provided more detailed insights into specific movement patterns such as subsidence or lift-off [7]. However, all mean rotations and translations are presented in the supplementary materials to give a clearer understanding of implant movements. Fourth, the study was underpowered to detect differences in PROMs. Hence, we focused solely on describing their changes since the 2-year period. For a clearer understanding of functionality scores associated with the uncemented ATTUNE TKR, observational studies with adequate statistical power, such as The ATTUNE Knee Outcome Study (ATKOS) [25], can provide more definitive insights.

### Conclusion

We showed that the ATTUNE femoral component exhibited significantly lower mean 5-year migration than the LCS femoral component, while this was comparable between tibial components. The mean 2- to 5-year migration rate and mean inducible displacement at 5 years were similar between the two TKR components and there was no difference in PROMs.

From a clinical perspective, the mean migration metrics suggest that the uncemented cruciate-sacrificing rotating platform ATTUNE has an equivalent long-term fixation compared with the LCS knee system.

### Supplementary data

Tables S1–S5 and Figure S1–S4 are available as supplementary data on the article page, doi: 10.2340/17453674.2024.42744

## Supplementary Material



## Notes

RP, BGP, and PAN conceived the follow-up study. RP, JS, and MS collected data from clinical patient visits. LAK collected and organized raw migration data. RP performed the data analyses. LAK, BGP, JS, BLK, and PAN helped interpret the results. RP wrote the manuscript.

The authors would like to thank MVR and DAV for performing surgery, and RR for the clinical assessments during the initial 2 years.

Handling co-editor: Per Henrik Randsborg

*Acta* thanks Sten Rasmussen and Stefan Maximillian Röhrl for help with peer review of this manuscript.
