# Supplementary material for: 5-year migration and inducible displacement of the uncemented LCS and ATTUNE rotating platform knee systems: a secondary report of a randomized controlled RSA trial
Source: Acta Orthop. 2025 Jan 10;96:59–65. doi: 10.2340/17453674.2024.42744 (PMC11724477; doi:10.2340/17453674.2024.42744)
Supplement: Supplementary file 1 [file ActaO-96-42744-s1.pdf]

Table S1. RSA REPORTING CHECKLIST

Serving as a checklist table specifically for prosthesis migration studies as an addition to the CONSORT guidelines

Section	Checklist item	Page
**Title**<br>**Abstract**	1. Identification as a radiostereometric analysis (RSA) study in title, abstract and keywords	1, 2, 3
**Methods**		
Study details	1. Report papers/references where prior results or partial results can be found	5<br>5
	2. First and last inclusion. Location and surgery information.	
	3. Number of surgeons that performed the surgeries.	5
Study groups	1. Detailed descriptions of prosthesis, cementation, and liner characteristics for each study group.	5
Follow-up	1. Information on weightbearing before or after first postoperative examination.	6<br>Previous
	2. Mean number and SD of postoperative time and baseline RSA examinations.	study<br>9
	3. Mean number and SD of postoperative time and endpoint RSA examination.	
RSA technique	1. Migration measurement method.	6
	2. Patient position (supine, weightbearing).	
	3. Software used, including version number.	6
	4. Location and orientation of the migration coordinate system.	6
	5. Use of fiducial/reference points to calculate MTPM.	6<br>n/a
Marker/model-based RSA technique	1. Image resolution (DPI) and type (CR, DR, film) or X-ray detectors.	Previous
	2. Material and size of markers.	study
	3. Calibration cage used, including type (uniplanar, bi-planar).	6
	4. Cutoff values for condition number and mean error of rigid-body fitting.	7
	5. Consistent or alternating X-ray method for RSA analysis.	6
**Results**		
Study flow	1. Number of migration examinations at different follow-up moments.	8
	2. Number and reasons why migration examinations were missing or excluded.	Figure 1.
Outcome	1. All migration data should be presented in millimeters and degrees.	Suppl file;
	2. Double examinations, mean, SD, and n for all outcome variables in the study.	Table A.<br>Table 1.
	3. Mean and SD of number of markers, CN and mean error of rigid-body fitting for each rigid body at the primary follow-up timepoint.	9
	1. Unmodelled results: mean, n and CI for each study group and follow-up timepoint presented in a table or figure or both.	Suppl file;<br>Table A.
Revision/failures	1. Number of prosthesis revision/failures in each treatment group.	9
	2. Migration values at last follow-up before revision or failure.	9

RSA: Radiostereometric analysis; CT: Computed tomography; DPI: Dots per inch; CR: Computed radiography; DR: Digital radiography; SD: Standard deviation; n: Number (of measurements); MTPM: Maximum total point motion; CI: 95% confidence interval.

Table S2. The mean migration and corresponding 95% confidence intervals of the uncemented Attune and LCS total knee replacements, as derived from the linear mixed-effects model

Follow-up, months	Tx, mm	Ty, mm	Tz, mm	Rx, °	Ry, °	Rz, °	MTPM. mm
**Attune femoral component**							
3	0.03 (–0.07 to 0.14)	0.17 (0.08 to 0.27) [a]	0.03 (–0.18 to 0.24)	–0.02 (–0.28 to 0.24)	–0.19 (–0.39 to 0.02)	–0.03 (–0.25 to 0.20)	0.64 (0.48 to 0.83) [a]
6	0.05 (–0.06 to 0.15)	0.19 (0.09 to 0.28) [a]	0.05 (–0.16 to 0.26)	–0.01 (–0.27 to 0.26)	–0.24 (–0.44 to –0.04)	–0.02 (–0.24 to 0.21)	0.76 (0.59 to 0.95) [a]
12	0.06 (–0.05 to 0.17)	0.22 (0.12 to 0.32) [a]	0.06 (–0.15 to 0.27)	–0.01 (–0.27 to 0.25)	–0.25 (–0.45 to –0.05)	0.01 (–0.21 to 0.23)	0.87 (0.78 to 1.07) [a]
24	0.05 (–0.05 to 0.16)	0.25 (0.15 to 0.34) [a]	0.04 (–0.17 to 0.25)	0.05 (–0.21 to 0.31)	–0.23 (–0.43 to –0.03)	0.01 (–0.22 to 0.23)	0.97 (0.78 to 1.19) [a]
60	0.11 (–0.01 to 0.22)	0.27 (0.17 to 0.37) [a]	0.02 (–0.19 to 0.24)	0.10 (–0.18 to 0.37)	–0.24 (–0.45 to –0.03) [a]	0.10 (–0.13 to 0.34)	1.14 (0.92 to 1.39) [a]
**LCS femoral component**							
3	0.01 (–0.01 to 0.12)	0.51 (0.41 to 0.61) [a]	0.10 (–0.11 to 0.32)	–0.02 (–0.49 to 0.05)	–0.34 (–0.55 to –0.13)	0.26 (0.03 to 0.50)	1.29 (1.06 to 1.56) [a]
6	0.00 (–0.11 to 0.11)	0.60 (0.51 to 0.70) [a]	0.19 (–0.02 to 0.41)	–0.38 (–0.70 to –0.11)	–0.43 (–0.64 to –0.22)	0.26 (0.03 to 0.49)	1.48 (1.22 to 1.76) [a]
12	0.00 (–0.10 to 0.12)	0.68 (0.58 to 0.77) [a]	0.18 (–0.04 to 0.40)	–0.46 (–0.73 to –0.19)	–0.61 (–0.82 to –0.40)	0.18 (–0.05 to 0.41)	1.75 (1.47 to 2.06) [a]
24	0.02 (–0.09 to 0.13)	0.72 (0.63 to 0.82) [a]	0.18 (–0.04 to 0.40)	–0.51 (–0.79 to –0.24)	–0.55 (–0.76 to –0.35)	0.28 (–0.05 to 0.41)	1.70 (1.42 to 2.01) [a]
60	–0.00 (–0.11 to 0.12)	0.77 (0.67 to 0.88) [a]	0.08 (–0.15 to 0.30)	–0.35 (–0.63 to 0.07)	–0.70 (–0.92 to –0.49) [a]	0.12 (–0.12 to 0.36)	1.87 (1.57 to 2.21) [a]
**Attune tibial component**							
3	0.00 (–0.09 to 0.09)	–0.31 (–0.43 to –0.18)	0.07 (–0.06 to 0.19)	–0.46 (–0.74 to –0.17)	–0.01 (–0.19 to 0.17)	–0.01 (–0.16 to 0.15)	0.98 (0.81 to 1.16)
6	–0.01 (–0.10 to 0.08)	–0.34 (–0.47 to –0.22)	0.10 (–0.02 to 0.22)	–0.48 (–0.76 to –0.10)	0.03 (–0.15 to 0.21)	–0.04 (–0.19 to 0.12)	1.06 (0.89 to 1.25)
12	–0.02 (–0.11 to 0.07)	–0.32 (–0.45 to –0.19)	0.09 (–0.04 to 0.21)	–0.34 (–0.62 to 0.06)	0.00 (–0.18 to 0.18)	–0.06 (–0.22 to 0.10)	1.08 (0.90 to 1.27)
24	–0.04 (–0.13 to 0.07)	–0.31 (–0.48 to –0.18)	0.10 (–0.03 to 0.22)	–0.22 (–0.50 to 0.06)	–0.01 (–0.19 to 0.17)	–0.06 (–0.21 to 0.11)	1.10 (0.92 to 1.29)
60	–0.05 (–0.14 to 0.04)	–0.28 (–0.41 to –0.15)	0.08 (–0.04 to 0.21)	–0.15 (–0.44 to 0.15)	–0.08 (–0.27 to 0.11)	–0.09 (–0.25 to 0.08)	1.13 (0.94 to 1.33)
**LCS tibial component**							
3	–0.10 (–0.19 to –0.01)	–0.24 (–0.37 to –0.11)	–0.09 (–0.21 to 0.03)	–0.38 (–0.67 to –0.10)	–0.04 (–0.22 to 0.14)	0.03 (–0.13 to 0.18)	0.82 (0.67 to 0.99)
6	–0.13 (–0.22 to –0.04)	–0.23 (–0.36 to –0.11)	–0.13 (–0.25 to 0.00)	–0.49 (–0.77 to –0.21)	–0.09 (–0.27 to 0.09)	0.01 (–0.15 to 0.17)	1.00 (0.83 to 1.18)
12	–0.13 (–0.22 to –0.04)	–0.25 (–0.38 to –0.12)	–0.14 (–0.27 to –0.02)	–0.46 (–0.74 to –0.18)	–0.08 (–0.26 to 0.10)	–0.04 (–0.20 to 0.12)	1.08 (0.91 to 1.27)
24	–0.14 (–0.23 to –0.05)	–0.26 (–0.39 to –0.13)	–0.14 (–0.26 to –0.02)	–0.31 (–0.59 to –0.03)	–0.17 (–0.35 to 0.01)	–0.12 (–0.28 to 0.04)	1.15 (0.97 to 1.35)
60	–0.15 (–0.24 to –0.06)	–0.21 (–0.34 to –0.07)	–0.13 (–0.25 to 0.00)	–0.36 (–0.65 to –0.06)	–0.40 (–0.59 to –0.21)	–0.20 (–0.36 to –0.04)	1.24 (1.05 to 1.46)

Rx, Ry, and Rz represent rotations around, and Tx, Ty, and Tz represent translations along the x-, y-, and z-axes. For a right-sided knee, positive translations are medial (x), proximal (y), and anterior (z) movements; positive rotations (°) are anterior tilt about the x-axis, internal rotation about the y-axis, and varus rotation about the z-axis. CI confidence interval; LCS Low Contact Stress; MTPM maximal total point motion.

[a] Parameters that are different between the 2 designs based on 95% confidence intervals.

Table S3. Inducible displacement and measurement error at 5 years postoperative, between the Attune and LCS components. Values are mean (95% confidence interval)

	Knees	Inducible displacement	Measurement error at 5 years (supine doubles)
**Femoral component**			
Attune	24		
Tx, mm		–0.03 (–0.09 to 0.02)	0.04 (–0.03 to 0.11)
Ty, mm		0.03 (0.01 to 0.04)	–0.01 (–0.03 to 0.01)
Tz, mm		–0.02 (–0.07 to 0.03)	–0.01 (–0.05 to 0.02)
Rx, °		0.01 (–0.05 to 0.08)	0.09 (0.00 to 0.19)
Ry, °		–0.04 (–0.12 to 0.04)	–0.02 (–0.09 to 0.04)
Rz, °		–0.07 (–0.15 to 0.01)	–0.02 (–0.07 to 0.02)
MTPM, mm		0.28 (0.21 to 0.35)	0.25 (0.17 to 0.30)
LCS	22		
Tx, mm		0.01 (–0.03 to 0.05)	–0.02 (–0.04 to 0.01)
Ty, mm		–0.01 (–0.05 to 0.03)	–0.01 (–0.04 to 0.02)
Tz, mm		–0.03 (–0.06 to –0.01)	0.03 (–0.01 to 0.08)
Rx, °		0.03 (–0.06 to 0.11)	–0.04 (–0.10 to 0.01)
Ry, °		–0.02 (–0.10 to 0.07)	0.12 (0.00 to 0.24)
Rz, °		–0.06 (–0.14 to 0.01)	0.01 (–0.07 to 0.10)
MTPM, mm		0.30 (0.23 to 0.36)	0.28 (0.18 to 0.36)
**Tibial component**			
Attune	24		
Tx, mm		–0.04 (–0.08 to 0.00)	–0.05 (–0.14 to 0.03)
Ty, mm		–0.05 (–0.07 to –0.03)	–0.02 (–0.05 to 0.00)
Tz, mm		–0.01 (–0.05 to 0.03)	–0.01 (–0.02 to 0.01)
Rx, °		–0.01 (–0.1 to 0.11)	–0.01 (–0.06 to 0.41)
Ry, °		0.20 (–0.21 to 0.62)	0.04 (–0.06 to 0.14)
Rz, °		0.04 (–0.03 to 0.12)	0.07 (–0.04 to 0.18)
MTPM, mm		0.37 (0.22 to 0.53)	0.28 (0.16 to 0.32)
LCS	24		
Tx, mm		–0.02 (–0.06 to 0.02)	–0.01 (–0.03 to 0.01)
Ty, mm		–0.04 (–0.09 to 0.01)	–0.01 (–0.03 to 0.01)
Tz, mm		–0.01 (–0.05 to 0.03)	0.02 (–0.01 to 0.04)
Rx, °		0.03 (–0.09 to 0.14)	–0.03 (–0.12 to 0.05)
Ry, °		0.05 (–0.15 to 0.24)	–0.06 (–0.21 to 0.09)
Rz, °		0.05 (–0.12 to 0.22)	0.02 (–0.01 to 0.05)
MTPM, mm		0.39 (0.29 to 0.51)	0.30 (0.25 to 0.35)

Rx, Ry, and Rz represent rotations around, and Tx, Ty, and Tz represent translations along the x-, y-, and z-axes. For a right-sided knee, positive translations are medial (x), proximal (y), and anterior (z) movements; positive rotations (°) are anterior tilt about the x-axis, internal rotation about the y-axis, and varus rotation about the z-axis.
LCS Low Contact Stress; MTPM maximal total point motion; CI confidence interval.

Table S4. Functional outcome measures of the patients with an uncemented Attune and LCS implant at 5-years postoperative

	Attune	LCS
Number of knees	24	24
Mean ROM, ° (SD)	123.8 (5.9)	120.0 (8.6)
Anteroposterior stability, n (%)		
< 5 mm	24 (100)	25 (100)
5–10 mm	0 (0.0)	0 (0.0)
> 10 mm	0 (0.0)	0 (0.0)
Mediolateral stability, n (%)		
< 5 °	20 (83)	20 (80)
5–9 °	4 (17)	4 (16)
10–14 °	0 (0.0)	1 (4.0)
> 14 °	0 (0.0)	0 (0.0)

ROM Range of motion; mm millimeter

Table S5. Scores of patient-reported outcome measures of patients with an uncemented Attune (n = 24) and LCS (n = 24) implant at 5-years postoperative

	Mean (SD)		Median (IQR)	
	Attune	LCS	Attune	LCS
Oxford Knee score	41.6 (7.0)	41.5 (7.6)	44.0 (37.3–47.8)	44.0 (39.0–47.0)
KOOS-PS	21.3 (14.9)	21.8 (17.8)	22.0 (10.5–32.7)	18.6 (5.6–36.6)
KUJALA	76.9 (15.3)	79.9 (17.6)	76.0 (68.0–88.0)	82.0 (70.8–97.3)
EQ-5D-3L	0.9 (0.1)	0.9 (0.2)	1.0 (0.8–1.0)	1.0 (0.8–1.0)
NRS at rest	1.0 (1.9)	1.2 (2.5)	0.0 (0.0–1.0)	0.0 (0.0–2.0)
NRS during movement	1.5 (2.1)	1.4 (2.5)	0.5 (0.0–2.8)	0.0 (0.0–2.0)

KOOS-PS; Knee injury and Osteoarthritis Outcome Score Physical Function shortform, KUJALA; anterior Knee Pain Scale.

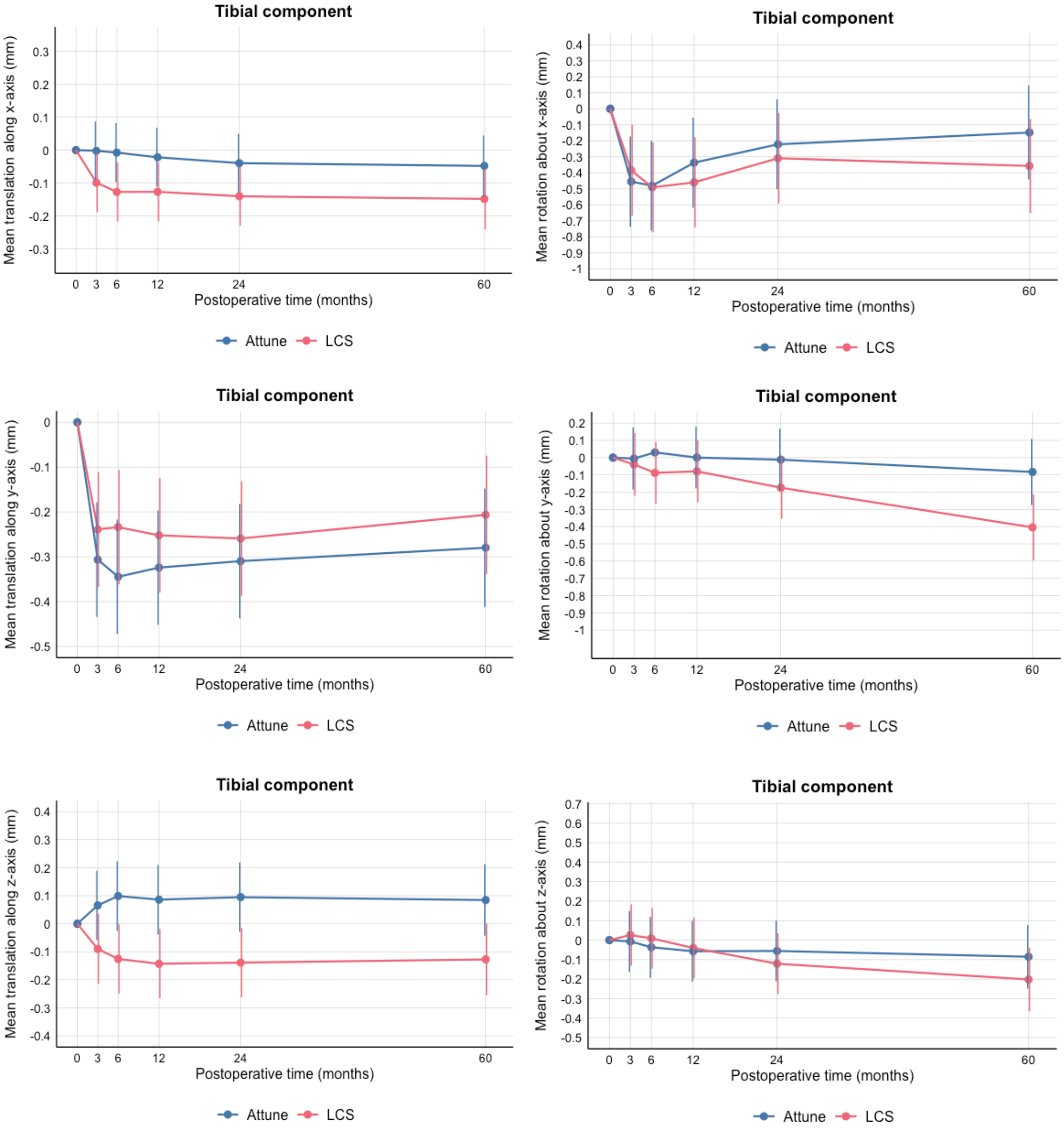

Figure S1. 5-year migration patterns of translations and rotations of the Attune and LCS tibial components.

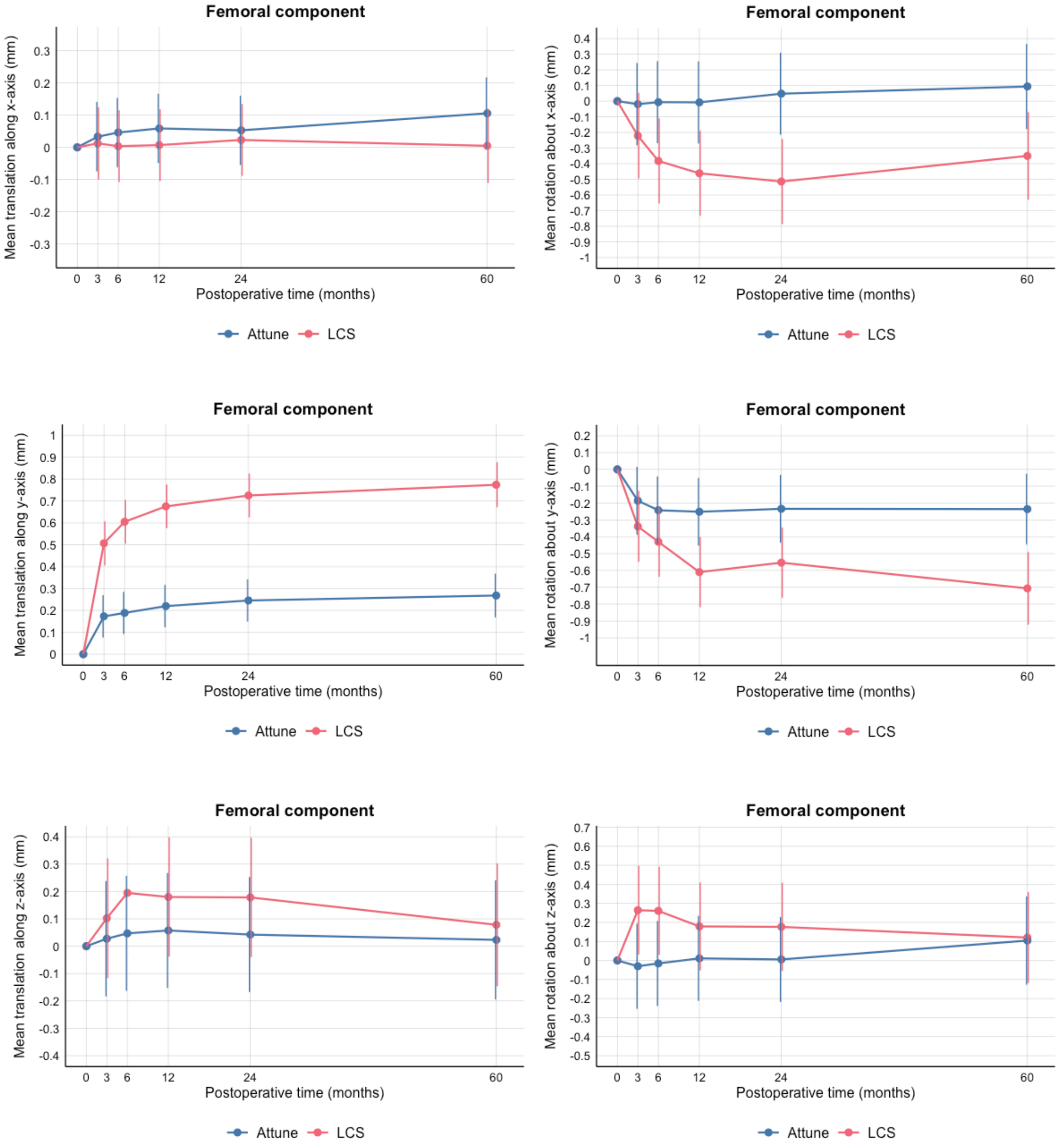

Figure S2. 5-year migration patterns of translations and rotations of the Attune and LCS femoral components.

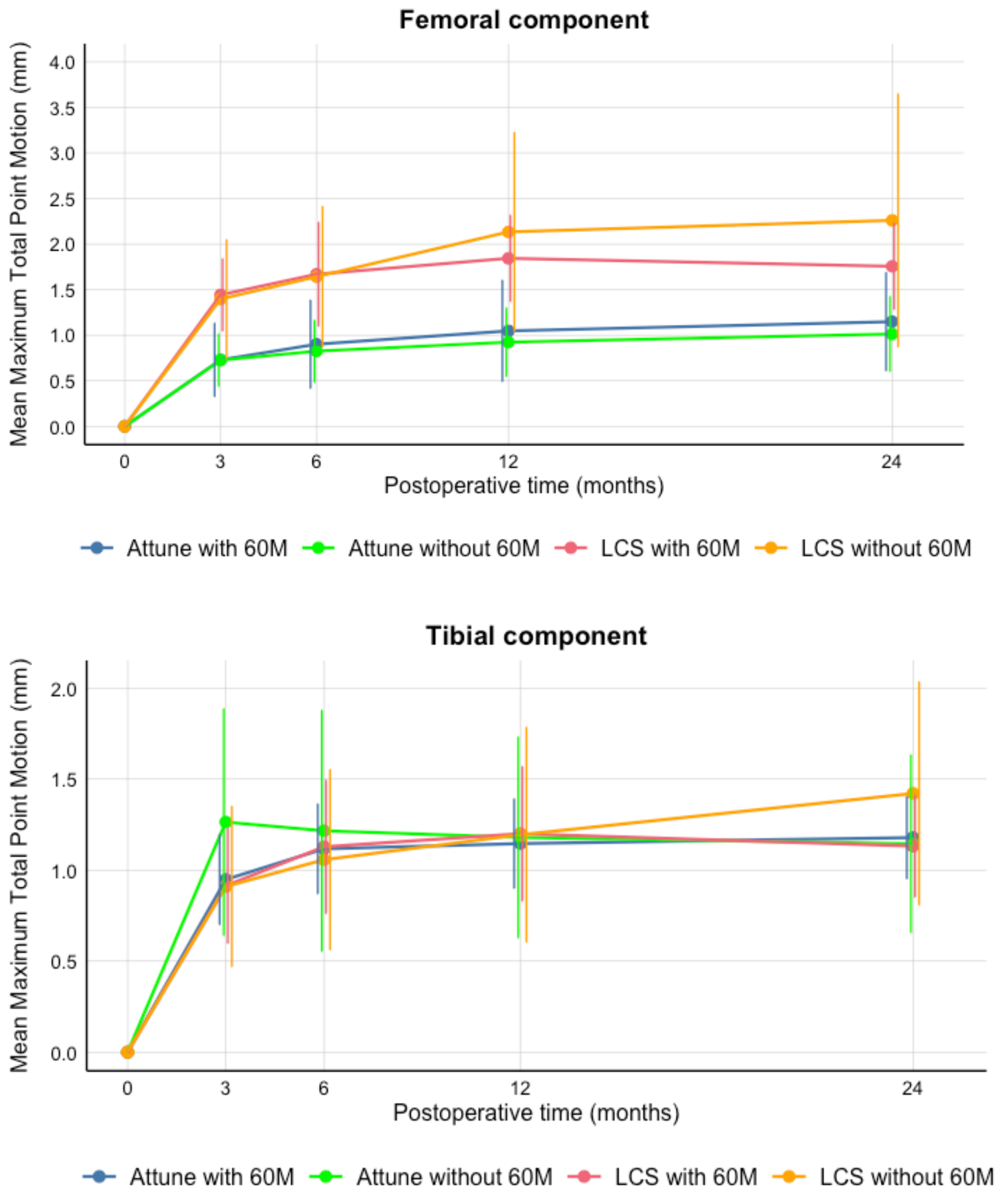

Figure S3. 2-year migration patterns of maximum total point motion of the Attune and LCS tibial and femoral components, concerning patients with and without 5-year migration data.

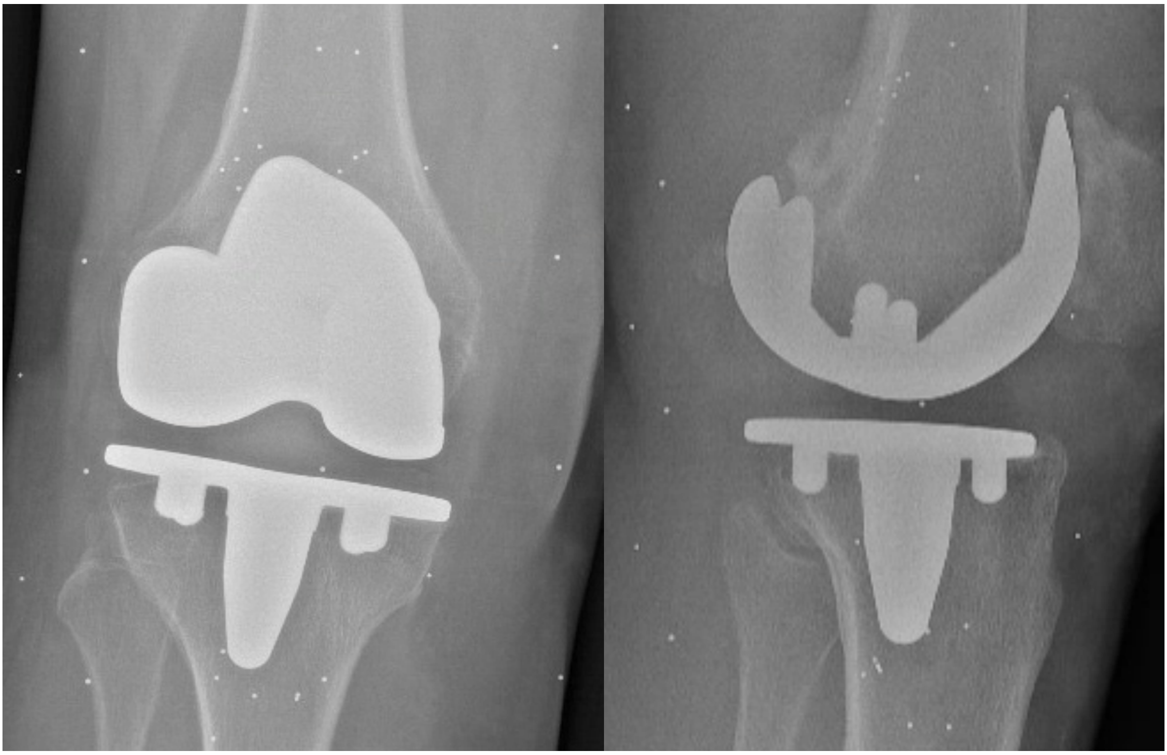

Figure S4. Radiostereometric radiographs of the ATTUNE total knee replacement with an inducible external rotation of 4.73° and 3.34 mm maximum total point motion. Both radiographs show a focal osteolysis on the lateral and posterior side.
